# Single-Position Lateral Lumbar Interbody Fusion Using Robotically Assisted Percutaneous Pedicle Screw Fixation in the Lateral Decubitus Position: Preliminary Results and Nuances of an Emerging Technology

**DOI:** 10.7759/cureus.107477

**Published:** 2026-04-21

**Authors:** Komal Naeem, S. Harrison Farber, Malika Bhargava, Randall W Porter

**Affiliations:** 1 Department of Neurosurgery, Barrow Neurological Institute, St. Joseph's Hospital and Medical Center, Phoenix, USA

**Keywords:** ct navigation, lateral lumbar interbody fusion, minimally invasive surgery, posterior fixation, robotically assisted surgery, single-position lateral

## Abstract

Introduction: This study reports a single-institution experience with robotically assisted single-position surgery for pedicle screw fixation and lateral lumbar interbody fusion in the lateral decubitus position (SL-ROB). Treatment outcomes, screw accuracy, and safety profile are discussed and compared with those of computed tomography-navigated surgery (SL-NAV).

Methods: A retrospective analysis of prospectively collected data was performed for all patients who underwent SL-ROB and SL-NAV from May 2018 to February 2020 at our institution (Barrow Neurological Institute, St. Joseph’s Hospital and Medical Center, Phoenix, AZ). Data on patient demographics, intraoperative details, the accuracy of screw placement, radiation dose, and outcomes were collected. Continuous variables were compared using the Mann-Whitney U test, and categorical variables were compared using the χ^2^ test.

Results: Seventeen patients (nine women and eight men) were enrolled. Ten underwent SL-ROB (mean (SD) age, 68.5 (7.5) years), and seven underwent SL-NAV (mean (SD) age, 59.0 (8.6) years). For the SL-ROB group, 11 levels were fused, and 40 pedicle screws were placed. The mean (SD) total operative duration, robot time, and time per screw were 4.6 (1.3) hours, 78 (48) minutes, and 7.0 (6.8) minutes, respectively. There were two instances of breach, and 32 of 34 (94%) screws had acceptable placement. Comparison of the SL-ROB and SL-NAV cohorts did not show significant differences in the mean operative time (p = 0.44) or treatment outcomes.

Conclusions: This series illustrates the efficiency and safety of SL-ROB. In comparison with SL-NAV, SL-ROB was associated with a similar operative time and improved screw placement accuracy. Operative efficiency improved over time in the SL-ROB cohort.

## Introduction

In recent decades, robotics has gained popularity in various surgical subspecialties, including neurosurgery. Robotic systems have been used in cranial surgery since 1985, when a robotic arm was first used to perform a brain biopsy using the basic principles of the Cartesian coordinate system [1]. With continued development since then, robotic systems are now commonly used for localization, orientation, and navigation in surgical cranial procedures [2]. However, the use of robotics in spine surgery is more recent, and the first case of posterior screw fixation using robotically assisted neuronavigation was reported in 2009 by Pechlivanis et al. [3]. The use of robotic navigation has been integrated with rapidly developing techniques for minimally invasive spinal surgery, where robotically assisted neuronavigation plays a critical role in safe and successful surgery. A recent meta-analysis reported higher accuracy for robotically navigated pedicle screw placement than for free-hand placement [4].

The minimally invasive lateral approach for interbody fusion, developed by Ozgur and colleagues in 2006 [5], minimizes tissue disruption and provides a larger implant footprint than posterior approaches. The lateral approach also provides indirect decompression of the neuroforamina. Performing lateral lumbar interbody fusion with posterior fixation traditionally necessitates a change in the patient's position from the lateral decubitus position to the prone position, which not only increases the operative duration and time under anesthesia but also involves risks associated with position change (e.g., risk of tube dislodgement or lead disconnection). Moreover, additional operating room staff may be needed during the change of position. Single-position surgery obviates the need for a change in position [6-8].

Computed tomography (CT)-navigated single-position surgery for pedicle screw fixation and lateral lumbar interbody fusion in the lateral decubitus position (SL-NAV) has already shown promising results, especially with respect to a reduction in operating room time [3,6-10]. Robotically assisted single-position surgery for pedicle screw fixation and lateral lumbar interbody fusion in the lateral decubitus position (SL-ROB) has also recently been described [11]. As robotically assisted spine fixation continues to become more popular, it is important to report experience with the technology and compare it with the existing technique to evaluate its efficiency and safety. In this study, we present our early experience with and evolving understanding of single-position surgery for pedicle screw fixation and lateral lumbar interbody fusion in the lateral decubitus position using robot navigation. We highlighted a single surgeon’s experience with this innovative technology. We explored the treatment outcomes of SL-ROB and compared them with those of SL-NAV.

This article was previously presented at the Society for Minimally Invasive Spine Surgery Annual Forum 2019 on November 1, 2019, at Las Vegas, Nevada.

## Materials and methods

Patient selection

We collected data for all consecutive patients who underwent SL-ROB and SL-NAV at our institution (Barrow Neurological Institute, St. Joseph’s Hospital and Medical Center, Phoenix, AZ) from May 2018 to February 2020, who were treated by the senior author (R.W.P.). Indications for surgery included patients with debilitating back pain with or without radiculopathy for whom conservative management had failed, who needed 1-2-level fusion, and who were good candidates for indirect decompression (i.e., did not require laminectomy or foraminotomy). We excluded all patients who had a staged (same-day or different-day) procedure. Approval was obtained from the Institutional Review Board of St. Joseph’s Hospital and Medical Center (PHX-18-0026-72-12), and the need for patient consent for study participation was waived.

Selection of appropriate patients is important for optimal outcomes of lateral position surgery, regardless of the method of neuronavigation used. The foremost surgical limitations include a high iliac crest causing access and approach difficulty, lean stature, and morbid obesity.

Screw placement planning

**Figure 1:**
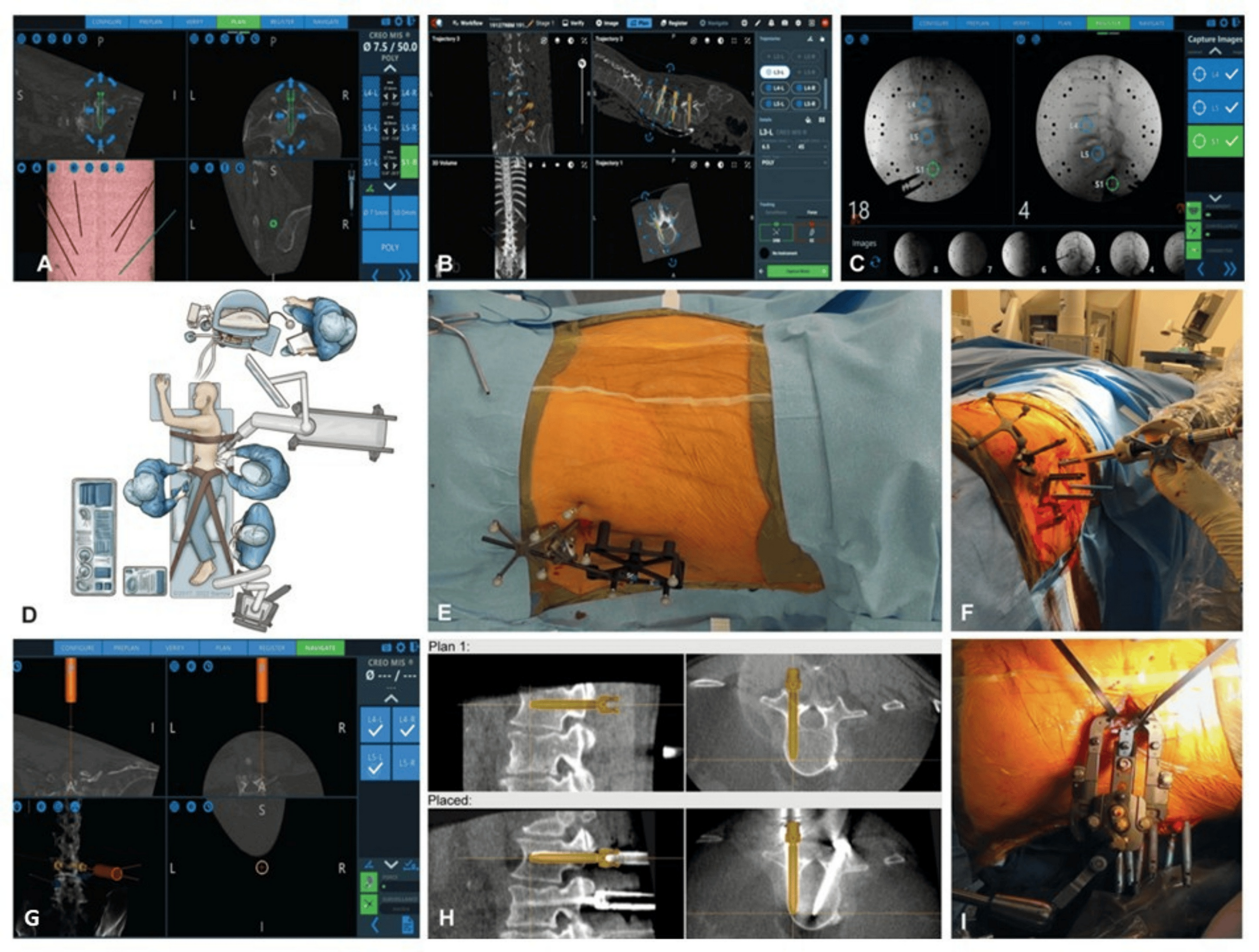
Screw planning, operating room set-up, and surgical procedures. (A-C) CT images are imported into screw planning software preoperatively, and placements are planned. (A) Screw plans (right S1) in the axial, coronal, and sagittal planes. Arrows along the screw allow fine adjustments. Image on the lower left shows trajectories of the robot arm; on the upper right, the estimation of screw size is shown. (B) Sagittal and coronal views of the entire screw trajectory plan. Vertebral levels are labelled, decreasing the risk of wrong-level surgery. (C) Fluoroscopic images used for registration are merged with preoperative CT images. Each level is registered separately. (D) Operating room set-up. The patient is positioned in the lateral decubitus position on the Jackson table with the left side up. The primary surgeon stands on the right side of the table during the entire procedure. The patient’s upper thorax and hips are taped to the table. The tape is crossed bilaterally over the left hip to stabilize the iliac crest, pull it down, and widen the working corridor. (E-I) Surgical procedure. (E) A dynamic reference base array and surveillance marker are placed on the posterior superior iliac spine and iliac crest, respectively, to aid with robot-assisted navigation (top view). (F) Robot arm position over the planned entry points (lateral view). (G) Simultaneous view of software, showing real-time orientation of the robot arm with respect to vertebrae. Notably, the trajectory is more vertical on the right side than on the left. (H) Sagittal and axial views of the planned trajectory of the right L1 vertebral body pedicle screw (top row). Post-procedure, sagittal and axial views of the actual placement of the pedicle screw of the right L1 vertebral body compared with the planned trajectory (golden overlay), showing the offset (bottom row). (I) Minimally invasive approach for interbody fusion (top view). Screw towers are left in place while performing interbody fusion. Used with permission from Barrow Neurological Institute, Phoenix, Arizona

In the SL-ROB group, preoperative or intraoperative CT was transferred to a robot guidance system (ExcelsiusGPS, Globus Medical, Audubon, PA) for screw trajectory planning following the three-dimensional rendering of the images. After the image quality was assessed, screw trajectories were planned (Fig. 1). For the SL-NAV cohort, a tracker was placed on the iliac crest, and intraoperative CT was performed. Screw trajectory planning then proceeded as described above for the SL-ROB cohort.

Patient positioning

Patients were positioned in the lateral decubitus position, with hips and knees flexed. For L4-L5 interbody fusion, the ipsilateral knee could be extended. An empty intravenous drip bag filled with air was placed underneath the left flank above the iliac crest to create downside flexion. When positioning patients for SL-ROB, it was critical to leave one hand’s width between the patient and the side of the bed, allowing better adjustment of the robotic arm, such that the edge of the bed did not interfere (Fig. 1). This positioning contrasts with that of SL-NAV, in which the patient is placed on the edge of the bed. The patient’s upper thorax and hips were taped down to avoid intraoperative shifting of their body, minimizing rotation of the bed and avoiding bumping the bed or tracker or excessive tapping (Fig. 1).

Surgical technique

Pedicle Screw Fixation

Robotically assisted pedicle screw fixation was done first, before interbody placement, to eliminate any shift in the patient’s body during fusion. Once the surgical field was prepped and draped, two small incisions were made on the posterior superior iliac spine and iliac crest to anchor the dynamic reference base array and surveillance marker (Fig. 1E). For robot guidance system registration, intraoperative fluoroscopic images were obtained and merged with CT images (obtained either preoperatively or intraoperatively). The system then calibrated the proposed screw trajectory, which can be refined by the primary surgeon. For SL-NAV, a neuronavigation system (Solera, Medtronic, Dublin, Ireland) was used for the pedicle screw fixation.

The robotic arm (ExcelsiusGPS) was brought into the surgical field and parked over each entry point. The skin and lumbodorsal fascia were incised with the blade using a guiding cannula attached to the robotic arm. The same guiding cannula was used for drilling the pedicles, tapping, and screw placement. The screws were placed using real-time neuronavigation, with towers in place (Fig 1F, 1G). Care was taken to avoid leaning on the drill while placing the screws to avoid a breach. Correct screw positioning can be confirmed with intraoperative fluoroscopy or CT. Rods were placed after interbody implant placement. On postoperative CT, the planned trajectory can be compared with the actual screw placement, and the offset can be calculated (Fig. 1H).

Lateral Lumbar Interbody Fusion

For the SL-ROB group, fluoroscopic images were obtained to ascertain the desired disc level. For the SL-NAV group, CT navigation was used. A 5-10-cm skin incision was made two-fingerbreadths' distance anterior to the desired disc level. Costectomy can be performed to improve exposure of higher-level disc spaces (e.g., L1/L2). After abdominal muscle blunt dissection, the retroperitoneum was entered, and the desired intervertebral disc was identified. The lumbar plexus was mapped using directionally triggered electromyographic stimulation. After the lateral vertebral body was reached, a cannulated corridor with a light source was established with free-run electromyography to avoid any nerve root injury, especially while placing the cannula (Fig. 1I). Subsequently, annulotomy was performed, and the disc space was cleaned. To prevent injury to the contralateral nerve exiting the foramina, it is critical to puncture the contralateral disc space only after cleaning the entire disc space. Once the interspace was sized, an implant of appropriate height, length, and lordosis was placed under fluoroscopic guidance. The cage was filled with demineralized bone matrix and bone morphogenetic protein. After copious irrigation and adequate hemostasis, a layered closure was performed.

Data collection

Demographic data were collected, including age, sex, body mass index (BMI, calculated as weight in kilograms divided by height in meters squared), diagnosis, operative time, estimated radiation dose, fluoroscopy time, and postoperative outcome. For the robotically assisted cohort, the time required for each step in the operation, including robot time (from the tracker incision on the posterior superior iliac spine to screw placement), was also recorded [12].

Postoperative CT was used to assess screw accuracy. Using the Spitz classification, deviation was measured in millimeters in case of any breach and classified as A (entirely within the pedicle), B (<2 mm), C (2-4 mm), and D (>4 mm) [13]. Placement within the pedicle and breach of <2 mm were considered acceptable screw placement.

Theory and calculation

We hypothesized that the learning curve for SL-ROB was steep and that the duration of the surgery would decrease and patient outcomes improve as surgeons gained experience. Descriptive analyses for the SL-ROB cohort were done, including calculations of mean or median values for continuous variables, and frequencies and percentages for discrete variables. Parameters for SL-ROB and SL-NAV were compared to assess whether the outcomes were similar to those associated with the existing technology. Continuous variables were compared using the Mann-Whitney U test, and categorical variables were compared using the χ^2^ test. A p-value ≤0.05 was considered significant. Variables with missing data were excluded from the analysis.

## Results

Demographic characteristics

The study included a total of 17 patients, 10 in the SL-ROB cohort and seven in the SL-NAV cohort. In the SL-ROB cohort, the mean (SD) age was 68.4 (7.5) years (range, 49-75 years), and six were male while four were female. The mean (SD) BMI in the SL-ROB cohort was 30.75 (3.72), classified as obese class I (range, 26.36-37.80) (Table 1) [13]. In total, 11 levels between L1 and S1 were operated on, and 40 pedicle screws were placed. All but one patient in the SL-ROB cohort had single-level fusion, and L4-L5 was the most common level requiring operation. Degenerative spondylosis was the most frequent preoperative diagnosis (four of 10 patients) in the SL-ROB cohort (Table 1).

**Table 1:** Demographic characteristics and intraoperative and postoperative details of patients treated with single-position lateral surgery for pedicle screw fixation and interbody fusion using robotically assisted neuronavigation. Data are no. (%) of patients, unless otherwise indicated. Abbreviations: BMI, body mass index, calculated as weight in kilograms divided by the square of height in meters; CT, computed tomography; ICT, intraoperative computed tomography; PGY, postgraduate year *Determined using the Spitz classification [13]. Postoperative CT was unavailable for some screws.

Parameters	Patients (n = 10)
Age (y), mean (SD)	68.5 (7.5)
Sex	
Male	6 (60)
Female	4 (40)
BMI, mean (SD)	30.75 (3.72)
Previous lumbar surgery	3 (30)
Levels	
Total	11
L1-L2	1 (9)
L2-L3	2 (18)
L3-L4	0 (0)
L4-L5	7 (64)
L5-S1	1 (9)
No. of levels fused	
Single	9 (90)
Double	1 (10)
Preoperative diagnosis	
Degenerative spondylosis	4 (40)
Spinal canal stenosis	3 (30)
Degenerative disc disease	3 (30)
Adjacent-level disease	0
Spine instability	7 (70)
Surgical duration (h), mean (SD)	
Overall	4.6 (1.3)
With preoperative CT	3.5 (0.3)
With ICT	5.4 (1.0)
Duration (min), median (IQR)	
Robot time	78 (65–113)
Tracker placement and screw planning	42 (33–61)
Screw fixation	32 (17–73)
Time per screw	7.0 (5.3–12.2)
Estimated CT* radiation dose (mGy), mean (SD)	70.52 (50.06)
Fluoroscopy time (s), mean (SD)	144.2 (70.2)
Screw repositioning, proportion (%) of screws placed	4/40 (10)
Estimated blood loss (mL), mean (SD)	197.5 (112.1)
Intraoperative complications	0 (0)
Length of stay (d), mean (SD)	4.6 (2.7)
Hip flexor weakness	
Immediate postoperative period	1 (10)
Six-week follow-up	2 (20)
Three-month follow-up	0
Groin or anterior thigh hypoesthesia	0
Screw accuracy,* proportion (%) of screws placed	
Acceptable (A, entirely within pedicle or B, <2 mm breach)	32/34 (94)
Breach (C, 2–4 mm or D, >4 mm)	2/34 (5.9); medial and lateral
Assisting surgeon	
Junior resident (PGY1–PGY3)	6 (60)
Senior resident (PGY4–PGY7)	4 (40)

**Figure 2:**
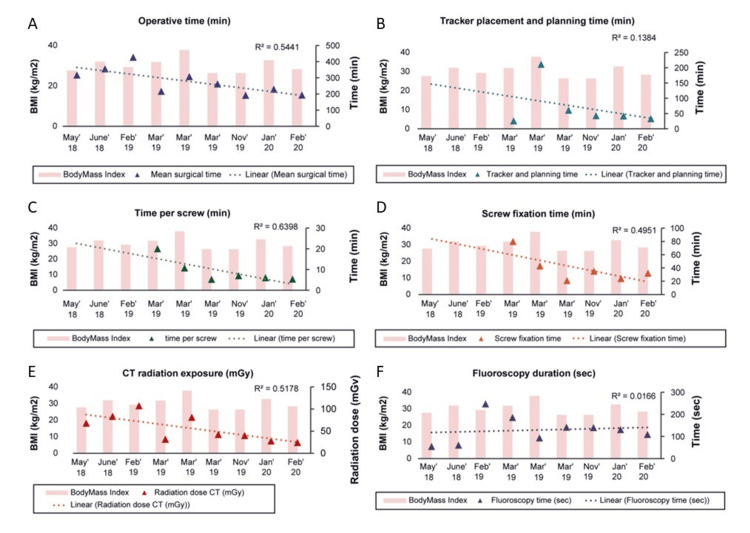
Graphic representations of operative and procedural times, radiation exposures, and correlations for all single-level fusion cases treated with robotically assisted single-position surgery for pedicle screw fixation and lateral lumbar interbody fusion in the lateral decubitus position from May 2018 to February 2020. (A) Operative time. (B) Mean tracker placement and planning time. (C) Mean time per screw. (D) Mean screw fixation time. (E) Mean CT radiation dose. (F) Mean fluoroscopy time. Abbreviations: BMI, body mass index; CT, computed tomography; min, minutes; mGy, milligray; sec, seconds Case 1, May 2018; case 2, June 2018; case 3, February 2019; cases 4-6, March 2019; case 7, November 2019; case 8, January 2020; case 9, February 2020. Used with permission from Barrow Neurological Institute, Phoenix, Arizona

In the SL-ROB cohort, the mean (SD) operative time was calculated as 4.6 (1.3) hours (range, 3.2-7.1 hours). For the patients who had preoperative CT, the operative time was significantly lower than that of those who underwent intraoperative CT (ICT) (3.5 (0.3) hours vs. 5.4 (1.0) hours; p = 0.006) (Table 1). The median (interquartile range (IQR)) robot time was 78 (65-113) minutes; robot time was divided into tracker placement and screw trajectory planning (median (IQR), 42 (33-61) minutes) and screw fixation (median (IQR), 32 (17-73) minutes). The median (IQR) time per screw was 7.0 (5.3-12.2) minutes (Fig. 2, Table 1). The mean (SD) CT radiation dose was calculated as 70.52 (50.06) mGy, and fluoroscopy time was 144.2 (70.2) sec. Four of 40 screws were repositioned, and a mean (SD) estimated blood loss of 197.5 (112.1) mL was reported. No intraoperative complications, including vascular or visceral injury, were reported. Postoperative imaging revealed that 32 of 34 (94%) screws had satisfactory placement, with a breach rate of 6%. Interestingly, a similar proportion of junior (postgraduate year [PGY] 1-3) and senior (PGY4-7) neurosurgery residents were surgical first assistants (60% and 40%, respectively) (Table 1).

During clinical follow-up, three patients reported hip flexor weakness (defined as motor power of four or less) between immediate and six-week follow-up. Weakness resolved for all patients by the three-month postoperative follow-up visit. None of the patients reported groin or anterior thigh hypoesthesia (Table 1).

SL-ROB trends

A case-by-case analysis was performed to determine the trends for mean surgical time, robot time, screw fixation time, and radiation exposure for all SL-ROB cases. Over the study period, a reduction of operative time was seen (r = -0.7; p = 0.02). Subanalyses of the robot time, screw fixation time, and time per screw showed decreases; however, the correlation coefficient did not reach statistical significance (r = -0.46, p = 0.18; r = -0.48, p = 0.17; and r = -0.58, p = 0.1, respectively). A comparison of the time per screw for the first three cases to that for the next four cases found a mean reduction of approximately eight minutes. Similarly, the total radiation exposure from CT decreased as more cases were performed (r = -0.7, p = 0.02) (Fig. 2).

Challenges encountered and their solutions

A critical review of each case was a mandatory step in our workflow. This aided in optimization. The procedure continued to be refined as the surgeon gained more experience. During the review, the challenges encountered and ways to avoid them in the future were discussed. Our experience may serve as a blueprint for other centers that are initiating robotic spine surgery programs. Below, we list the challenges that were encountered with the introduction of SL-ROB and how those challenges were addressed (Table 2) [13].

**Table 2:** Descriptive analysis of 10 patients treated with single-position pedicle screw fixation and lateral interbody fusion using robotically assisted neuronavigation. Abbreviations: Est., estimated; ICT, intraoperative computed tomography; K-wire, Kirschner wire; N/A, not applicable; ND, no data Spitz classification for screw accuracy (breach): A (entirely within pedicle), B (<2 mm), C (2–4 mm), or D (>4 mm) [13]. *Screw trajectory planning was performed on preoperative computed tomography. A single mention of “A” signifies that all screws had a classification of A.

Patient	Age (y), sex	No. of screws; Spitz accuracy	Level fused	Operative time (min)			Time per screw (min)	Est. radiation dose (mGy)	Fluor­oscopy time (s)	Intraoperative problems encountered	Solutions
				Total	Robot time						
					Tracker placement and planning	Screw fixation (first screw incision to last screw in)					
1	75, M	2; ND	L5-S1	317	ND	ND	ND	68	55	None	N/A
2	74, M	4; A	L2-L3	355	ND	ND	ND	84	60	None	N/A
3	70, F	4; A, A B, A	L4-L5	427	ND	ND	ND	107	247	Entire plan shifted because of the repositioning of the patient after acquiring the ICT; the left L4 and L5 screw were malpositioned due to drill skiving laterally for left L4 and L5.	Performed the ICT again; L4 and L5 screws were repositioned using fluoroscopy.
4	73, M	6; A	L1/L2, L2/L3	277	40	73	12	198	278	Auto planning of two screws was not appropriate; screw malposition due to drill skiving; left L1-medial; right L2-lateral.	ICT was repeated for the planning; screws were repositioned using fluoroscope; the drill tip was changed for future operations.
5	65, F	4; ND	L4/L5	216	26*	80	20	32	186	Concerns about the medial right L5 breach after screw planning	K-wire was placed in the pedicle; positioning was ascertained with fluoroscopic guidance.
6	49, M	4; C, A A, A	L4/L5	307	211	43	11	81	93	ICT was off-center.	Rotated 15° off the center
7	71, F	4; A	L4/L5	262	61	17	5	43	142	Lean body build causing difficulty in accessing interbody laterally	Required patient repositioning
8	71, M	2; A	L4/L5 (unilateral fixation)	192	43*	14	7	40	140	None	N/A
9	67, F	4; A	L4/L5	229	42*	24	6	28	131	Difficulty in the positioning of the robot arm	Robot arm was rehomed, and software was restarted.
10	70, M	6; A	L4/L5	193	33*	32	5	24	109	None	N/A

Shift in the Position

Optimal positioning plays a critical role in attaining high screw accuracy. The surgeon should be attentive to any shift in the patient’s position and refrain from performing any maneuvers that can lead to shifting, like excessive tapping.

Skiving

During drilling of the downside pedicle, the drill bit skived due to the relatively vertical trajectory. This was resolved by replacing the straight drill with a round drill burr during the initial part of the drilling. The round ball tip, which has a higher number of revolutions per minute and lower torque than the straight drill, creates a pilot hole for the straight drill, making skiving less likely. This technique eliminated the incidence of skiving in the remainder of the cases (Table 2).

Acquiring Preoperative CT

We experienced a significant reduction in mean (SD) surgical duration with the implementation of a preoperative CT workflow, compared with an ICT workflow (3.5 (0.3) vs. 5.4 (1.0) hours, p = 0.006).

Screw Planning

The workflow was modified with the preoperative acquisition of the CT scan, which allowed the screw trajectory to be planned before the operation. Initially, the trajectory was planned by an attending physician in the operating room while the patient was anesthetized and intubated. Altering this workflow also contributed to a reduction in the total operative duration.

Comparison of the SL-ROB and SL-NAV groups

In the SL-NAV cohort (seven patients), 32 screws were placed on nine levels between L2 and S1. The mean (SD) age was 59.0 (8.6) years (range, 41-66 years), and the mean (SD) BMI was 29.14 (7.13) (range, 23.08-43.55) (Table 1). Patients in the SL-ROB cohort were older than those in the SL-NAV cohort (p = 0.008). No significant difference in sex, BMI, or frequency of prior lumbar surgery was found between the two groups (Table 3) [13]. The duration of surgery was similar for both SL-ROB and SL-NAV (mean (SD) duration, 4.6 (1.3) hours vs. 4.2 (1.1) hours; p = 0.06). The mean CT radiation dose was similar (mean (SD) radiation, 70.52 (50.06) mGy vs. 42.06 (15.32) mGy; p = 0.09), whereas the mean fluoroscopy time was significantly higher for the SL-ROB cohort (144.2 (70.2) seconds vs. 26.3 (13.2) seconds; p = 0.001) (Table 3) [13].

**Table 3:** Comparison of the demographic characteristics and intraoperative and postoperative details of patients treated with single-position surgery for pedicle screw fixation and interbody fusion using robotically assisted neuronavigation (SL-ROB) versus computed tomography-navigated screw fixation (SL-NAV). Data are no. (%) of patients unless otherwise indicated. Boldface type indicates statistical significance. Continuous variables were compared using Mann-Whitney U test, and the χ2 test was used for the comparison of categorical variables. Abbreviations: BMI, body mass index, calculated as weight in kilograms divided by the square of height in meters; CT, computed tomography; ICT, intraoperative computed tomography *Determined using the Spitz classification [13]. Postoperative CT was unavailable for some screws.

Parameters	SL-ROB (n = 10)	SL-NAV (n = 7)	Test statistic	p-value
Age (y), mean (SD)	68.5 (7.5)	59.0 (8.6)	8.0	0.008
BMI, mean (SD)	30.75 (3.72)	29.14 (7.13)	20.0	0.14
Previous lumbar surgery	3 (30)	3 (43)	0.3	0.6
Total surgical duration (h), mean (SD)	4.6 (1.3)	4.2 (1.1)	10.0	0.06
Estimated CT radiation dose (mGy), mean (SD)	70.5 (50.1)	42.1 (15.3)	17.5	0.09
Fluoroscopy time (s), mean (SD)	144.2 (70.2)	26.3 (13.2)	0.00	0.001
Screw repositioning, proportion (%) of screws placed	4/40 (10)	1/25 (4)	0.78	0.38
Length of stay (d), mean (SD)	4.6 (2.7)	5.9 (2.7)	47.0	0.24
Hip flexor weakness				
Immediate postoperative period	1 (10)	2 (29)	0.98	0.34
Six-week follow-up	2 (20)	0	1.6	0.22
Three-month follow-up	0	0		
Groin or anterior thigh hypoesthesia				
Immediate postoperative period	0	1 (14)	1.52	0.23
Six-week follow-up	0	0		
Screw accuracy,* proportion (%) of screws placed				
A (entirely within the pedicle)	32/34 (94)	16/21 (76)	3.76	0.05
B (<2 mm)	1/34 (3); medial	2/21 (10); 1 lateral, 1 medial	1.09	0.30
C (2–4 mm)	1/34 (3); lateral	1/21 (5); lateral	0.12	0.72
D (>4 mm)	0	2/21 (10); lateral	3.36	0.07

There was no significant difference in the length of hospital stay for either group (p = 0.24). No intraoperative complications were reported for either group. The frequency of procedure-related postoperative complications was similar between the two groups. The proportion of screws with acceptable placement was higher in the SL-ROB group than in the SL-NAV group (94% vs. 76%; p = 0.05), whereas no significant difference in the frequency of screw repositioning was found (p = 0.38) (Table 3).

Illustrative case

**Figure 3:**
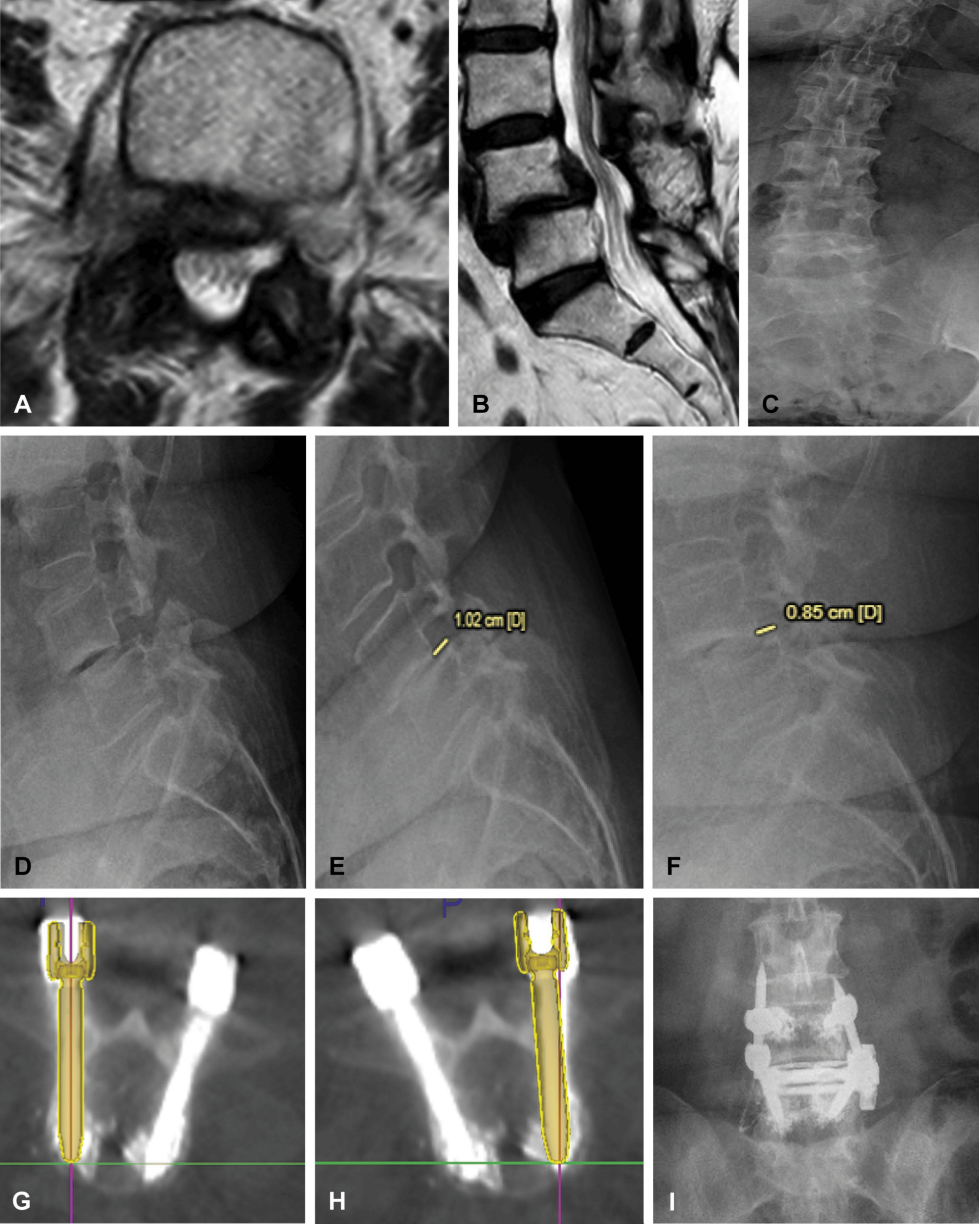
Case illustration of a woman in her late 60s presented with severe right hip and lateral thigh and shin pain. She had previously undergone a laminectomy at L4-L5. Axial (A) and sagittal (B) preoperative T2-weighted magnetic resonance imaging showing severe right foraminal stenosis, extrusion of the intervertebral disc, and grade II L4-L5 anterolisthesis. (C) Anteroposterior and (D) lateral views of standing radiographs showing dextro-convex curvature and L4-L5 anterolisthesis and bilateral pars defect. (E) Flexion and (F) extension radiographs showing reduction in slippage with extension. Postoperative axial views of planned and placed trajectory of (G) right and (H) left L4 vertebral body pedicle screw. Placed screws can be compared with the planned trajectory (golden overlay), and the offset can also be calculated. (I) Frontal view of postoperative radiograph ascertaining the placement of the pedicle screws and interbody. Used with permission from Barrow Neurological Institute, Phoenix, Arizona

A woman in her late 60s presented with severe right hip, lateral thigh, and shin pain. She previously had undergone a laminectomy at L4-L5. However, the symptoms were not completely resolved, and the pain continued to worsen. The pain was refractory to other conservative measures, like physiotherapy and epidural injections. Preoperative weight-bearing imaging revealed dextro-convex curvature and a grade II L4-L5 anterolisthesis and bilateral pars defect. Dynamic imaging with flexion and extension radiographs showed a reduction in slippage with extension (Fig. 3). Preoperative magnetic resonance imaging showed severe right foraminal stenosis, intervertebral disc protrusion, and anterolisthesis at L4-L5 (Fig. 3). The patient underwent single-position lateral L4-L5 interbody fusion and robotically assisted posterior screw fixation of L4 and L5 vertebrae. The patient reported an improvement in symptoms immediately after the surgery. Postoperative radiography showed a reduction in slippage, and CT showed that the placement of all screws was acceptable (Fig. 3). Postoperatively, the patient denied complaints of hip flexor weakness or hip hypoesthesia.

## Discussion

This study highlighted a single-surgeon experience with 10 initial cases treated with SL-ROB. We outlined the procedure details and reported satisfactory postoperative outcomes for our patient cohort. This cohort included older patients with multiple comorbid conditions, which supports the favorable safety profile of SL-ROB (Table 1).

Since the introduction of the lateral approach for interbody fusion in 2006 by Ozgur and colleagues [5], the technique has been widely studied, adapted, and modified [6-8,11]. However, performing pedicle screw fixation as a same-day procedure required a patient position change, which increased the operative time. Single-position surgery has proved to be an efficient solution to the problem. Data on single-position surgery have been promising, with favorable outcomes and accuracy [6,7,14-16]. The findings of this study have been similar.

In the past decade, there has been an exponential increase in the use of robotics in spine surgery. As we incorporate this novel technology into our practices, a common goal has been to reduce operative time and radiation exposure while improving the screw accuracy and treatment outcomes, when compared with other minimally invasive and open procedures. We report a mean (SD) operative time of 4.6 (1.3) hours, time per screw of 7.0 (6.8) minutes, and screw accuracy of 94% (32 of 34 screws). Our screw accuracy and time per screw findings are comparable to findings published elsewhere [3,12], whereas the mean operative duration was found to be longer than that reported in a study involving a larger cohort (55 patients; mean (SD) operative duration, 155.7 (42) minutes) [14]. However, we observed a wide range of operative durations in our study (3.2-7.12 hours), which is most likely attributable to the learning curve.

The literature shows that, with time and a continued increase in the sample size experience of the operating team, including the surgeon and operating room staff, surgical proficiency increases. With more experience, our operating time and the challenges encountered decreased, while we continued to have favorable outcomes. A case-to-case analysis revealed a decreasing trend in the operative time (r = -0.7, p = 0.02) and CT radiation exposure (r = -0.7, p = 0.02) over time. As suggested in other studies, our data show improvement with more experience (Fig. 2) [12,17].

With constant feedback, we refined the workflow and performed a post-hoc review for each case to assess whether these measures were associated with any improvements in performance or outcomes. We switched from the initially recommended intraoperative CT to preoperative CT and noticed a reduction in operative duration. The preoperative CT protocol led to a modification in screw trajectory planning. With the revised protocol, the planning was initially done by the resident preoperatively and was later refined by the attending physician. This change improved the workflow and created learning opportunities for the trainees. In addition, the mean (SD) surgical duration was 3.5 (0.3) hours with the implementation of the preoperative CT workflow compared with 5.4 (1.0) hours with the ICT workflow (p = 0.13). The reasons for this 35% difference in time are multifactorial, and experience-led improvement in the surgery team's skill set plays an essential role. We report a decrease of 8.4 minutes in our time per screw when the first three cases are compared with the next four cases. A similar trend has been identified in other studies, which have reported a 1.5-minute decrease between the first 15 and next 15 cases [18] and a 6.9-minute reduction between the first 14 and next 13 cases [19].

A recent meta-analysis concluded that the use of robotically assisted navigation to place pedicle screws was associated with better screw accuracy and decreased radiation exposure compared with the freehand technique. However, the placement of screws with robot navigation was found to be associated with longer operative times [4]. We made a similar comparison between robotically assisted and CT-guided navigation systems. We do not report any significant difference in the mean (SD) operative time between procedures performed with robotically assisted navigation (4.6 (1.3) hours) and those performed with CT neuronavigation (4.2 (1.1) hours; p = 0.06). It is difficult to make meaningful inferences about the difference in radiation exposure between the two groups because of the difference in technique. A significantly higher fluoroscopy time for the SL-ROB group is attributable to the use of fluoroscopy for the interbody fusion, as opposed to the use of CT navigation in the SL-NAV group (p = 0.001). We do not report any difference in the radiation exposure from CT between the two groups (p = 0.09).

Our study found that screw placement was more accurate in the SL-ROB group than in the SL-NAV group (p = 0.05). When interpreting the comparison between the navigation systems, it is critical to keep in mind that these were the first 10 cases of SL-ROB performed by the senior author (R.W.P.), whereas the surgeon was experienced with SL-NAV. The goal of comparison is to report that the SL-ROB outcomes and safety profile are comparable to those of the existing technology.

In the literature, the rate of vascular injuries associated with the lateral approach has been reported to be as high as 1.6% [15]. However, we do not report any intraoperative complications, including vascular injuries, for the SL-ROB or SL-NAV group. The immediate postoperative recovery period was also uneventful. All patients were discharged to home or an acute rehabilitation facility with a mean (SD) length of stay of 4.6 (2.7) days, and no significant difference was found between the two groups. Overall, minimally invasive surgery is associated with a shorter length of stay, compared with surgery performed with the use of the free-hand technique, because the former involves a smaller incision and less muscle manipulation [17,20]. A recent meta-analysis reported a cumulative incidence of 9.43% for transient hip flexor weakness in minimally invasive lateral interbody arthrodesis [16]. Similarly, an incidence of 3.88% is reported for hip hypoesthesia [16]. We report five patients with transient hip flexor weakness and one patient with groin or anterior thigh hypoesthesia in our cohort of 17 patients. Moreover, in the SL-ROB group, two patients had transient hip flexor weakness, and none had groin or anterior thigh numbness (Table 1).

Limitations of the study

The small sample size limits the generalizability of our findings. It also limits our ability to perform a comprehensive comparative analysis. In addition, due to the retrospective nature of the study, the selection of patients to undergo SL-NAV or SL-ROB cannot be randomized. This situation can be a possible source of bias. Therefore, additional prospective studies with larger sample sizes assessing long-term outcomes are required to establish that this novel technique is associated with better screw accuracy, less radiation exposure, and an improvement in treatment outcomes.

This study includes the initial cases treated with this technique, which required experience to gain expertise. The association of experience with expertise is evident in the wide range of surgical times and radiation doses, for which a single value (i.e., mean) might not be truly representative of the data.

## Conclusions

This study outlines the SL-ROB technique and describes the safety and efficacy of this novel technique. We report that SL-ROB not only has the advantage of not requiring a change in patient position but also can potentially decrease patients’ time under anesthesia. We report favorable treatment outcomes with the application of this innovation. The results were comparable to those achieved with the existing technology. It is critical to adopt novel technologies and provide feedback so that surgical techniques can be improved and procedures can be made more efficient and safer. Although this study is preliminary, with a small sample size, future prospective studies with larger sample sizes will provide better insights regarding improvement in long-term outcomes.
